# Efficient Generation of Recombinant Influenza A Viruses Employing a New Approach to Overcome the Genetic Instability of HA Segments

**DOI:** 10.1371/journal.pone.0116917

**Published:** 2015-01-23

**Authors:** Ahmed Mostafa, Pumaree Kanrai, Henning Petersen, Sherif Ibrahim, Silke Rautenschlein, Stephan Pleschka

**Affiliations:** 1 Center of Scientific Excellence for Influenza Viruses, National Research Center (NRC), Cairo, Egypt; 2 Institute of Medical Virology, Justus Liebig University Giessen, Giessen, Germany; 3 Clinic for Poultry, University of Veterinary Medicine Hannover, Hannover, Germany; 4 Department of genetic engineering, Veterinary Serum and Vaccines Research Institute (VSVRI), Agricultural Research Center (ARC), Cairo, Egypt; Icahn School of Medicine at Mount Sinai, UNITED STATES

## Abstract

Influenza A viruses (IAVs) are the most relevant and continual source of severe infectious respiratory complications in humans and different animal species, especially poultry. Therefore, an efficient vaccination that elicits protective and neutralizing antibodies against the viral hemagglutinin (HA) and neuraminidase (NA) is an important strategy to counter annual epidemics or occasional pandemics. With the help of plasmid-based reverse genetics technology, it is possible that IAV vaccine strains (IVVS) are rapidly generated. However, the genetic instability of some cloned HA-cDNAs after transformation into competent bacteria represents a major obstacle. Herein, we report efficient cloning strategies of different genetically volatile HA segments (H5- and H9-subtypes) employing either a newly constructed vector for reverse genetics (pMKP*ccd*B) or by the use of the *Escherichia coli* strain HB101. Both approaches represent improved and generalizable strategies to establish functional reverse genetics systems preventing genetic changes to the cloned (HA) segments of IAV facilitating more efficient rescue of recombinant IAV for basic research and vaccine development.

## Introduction

Influenza viruses, belonging to the family *Orthomyxoviridae,* are divided according to the antigenic difference between their nucleoprotein (NP) and matrix 1 (M1) proteins into three genera; A, B or C [1]. Out of these three genera, influenza A viruses (IAV) are the most likely to cause serious infections in different species including humans [2]. IAV is composed of eight negative sense genomic RNA segments within a lipid-bilayer envelope. According to the antigenicity of the two surface glycoproteins, hemagglutinin (HA) and neuraminidase (NA), IAV can be subtyped into 18 HA subtypes and 11 NA [3–7].

Avian IAVs affect not only poultry industries, but are also potential threats to human health [8]. In recent years, many avian and porcine IAV have crossed the host barrier resulting in sometimes fatal infections of humans, such as the pandemic swine-origin influenza virus (S-OIV) H1N1 in 2009, highly pathogenic avian influenza virus (HPAIV) H5N1 and low pathogenic avian influenza viruses (LPAIV) H7N9 and H10N8 [9–12]. For these reasons, rapid development of new or adjusted influenza virus vaccine strains (IVVS) is an important and efficient tool to protect both human and poultry against potential health- and economic threats. Here the development of, plasmid-based reverse genetics (RG) systems to generate IAV from cloned cDNA represents a major improvement [13–15]. The respective RNA-segments encoding HA- and NA, against which protective antibodies are being generated after infection/vaccination, can now be cloned into RG vectors and are then transfected into cells together with six additional RG plasmids encoding the remaining genome segments of A/Puerto Rico/8/34 (PR8, H1N1), which replicates to high titers in embryonated chicken eggs, allowing high yields of the respective IVVS [7,16,17] Ultimately, RG permits the rapid generation of recombinant IVVS with a desired genome composition to reduce the impact or even protect against influenza epidemics and pandemics [18–21]. However, eventual genetic instability of plasmids encoding HA or the NA-segments in *Escherichia coli (E. coli)* represents a major obstacle on the way to generate candidate IVVS.

Although plasmids use different systems/mechanisms to keep their genetic stability in *E. coli*, the instability of plasmids can be induced either by the random expression of toxic gene products, presence of AT-rich sequences, the plasmid copy number (low or high), secondary and tertiary structures, genotype of the competent cells as well as the presence of long repeats and promoters [22–28]. In *E. coli*, the multi-functional *RecA* protein is essential for most homologous recombination and repairing of the double-stranded DNA breaks [29,30]. To limit or inhibit the recombination of the transformed DNA with the *E. coli* genome, the *RecA* gene of different bacterial strains was mutated to generate different *rec*A alleles (e.g. *recA*1, *recA*13 and *recA* ^−^) [31,32]. Moreover, a low cultivation temperature and plasmids with a low copy origin of replication were also reported to be beneficial to maintain high plasmid stability [33–35].

Recently, the instability of plasmids encoding IAV genomic segments was reported for the PB2 segment of A/Turkey/Ontario/7732/1966 (H5N9) when cloned into common cloning plasmids like pcDNA3.1/V5-His-TOPO (Invitrogen, USA) or established RG plasmids like pHH21 [14] using different competent bacterial strains (*recA*1, *recA*13 and *recA* or *recB* deficient), temperatures (25°C, 30°C and 37°C) and antibiotic concentrations [36]. Here, we demonstrate how we overcame such difficulties in cloning the HA-cDNAs of two avian IAV from different subtypes, H9N2 (H9N2/SA, A/chicken/Saudia Arabia/CP7/98) and H5N1 (H5N1/VSVRI, A/chicken/Egypt/VSVRI/2009).

The genetic instability of the cDNA encoding HA segments of the mentioned strains in standard RG plasmids pHW2000 [37] and pHH21 was detected when regularly used *E. coli* strains were transformed (e.g. DH5α and Top10). Nevertheless, we could stably clone the HA-cDNAs by two different approaches: (1) by transformation of the HA-cDNAs cloned into the standard RG vector pHW2000 into the *recA13 E. coli* strain HB101 and (2) by cloning the unstable HA-cDNAs into the improved RG vector, pMKP*ccd*B, followed by transforming the plasmid DNA into the commonly used competent *E. coli* strains like DH5α and XL1-blue. These two approaches permitted us to generate prototype IVVS with the HA and NA of H9N2/SA and H5N1/VSVRI. For this, the HA- and NA-encoding plasmids based either on pHW2000 or pMKP*ccd*B were transfected together with pHW2000 plasmids encoding the remaining six internal genome segments of PR8. Both IVVS generated by these approaches showed comparable replication efficiency. We thereby demonstrate that these two cloning strategies provide a powerful tool to prevent genetic instability of cloned viral segments and simplify the generation of complete reverse genetics systems and vaccine strains for influenza A viruses.

## Materials and Methods

### Cells, viruses and viral cDNA

Madin-Darby canine- and human embryonic kidney cells, MDCK-II and 293T, were maintained in Dulbecco’s Modified Eagle Medium (DMEM) (Gibco, Invitrogen, USA) supplemented with 100 I.U./ml penicillin, 100 μg/ml streptomycin and 10% fetal bovine serum (FBS). All cells were incubated at 37°C in the presence of 5% CO_2_. Virus stock of the influenza virus A/chicken/Saudi Arabia/CP7/1998 (H9N2/SA) was obtained by inoculation of 100 μl (100 FFU) through allantoic route into 10-day-old embryonated chicken eggs. After 48 h incubation at 37°C in an egg incubator (Heka, Germany), eggs were chilled at 4°C overnight. The allantoic fluid was then harvested and centrifuged at 2500 rpm for 5 min to remove cell debris. The cDNA for A/chicken/Egypt/VSVRI/2009 (H5N1/VSVRI) was obtained from the Egyptian Veterinary Serum and Vaccine Research Institute (VSVRI).

### Full-length RT-PCR amplification of HA and NA segments

The viral RNA of the H9N2/SA virus was extracted using “RNeasy Protect Kit” (Invitrogen, USA) according to the manufacturer’s instructions. The HA and NA segments of H9N2/SA were then amplified in separate reactions using “SuperScript III One-Step RT-PCR Platinum Taq High Fidelity” (Invitrogen, USA). Briefly, 5μl of extracted vRNA was mixed with 25μl “2× Reaction Mix”, 2μl (0.4 μM each) of the Blunt-Ba-HA-F and Blunt-Ba-HA-R primers for the HA segment (Table 1) or universal primers [38] with *BsmB*I restriction sites for the NA segment (Table 1) of H9N2/SA and 1 μl of enzyme mix. The total volume was adjusted to 50 μl using nuclease-free water (Ambion, USA) and subjected to cDNA synthesis at 55°C for 30 min, followed by pre-denaturation (94°C for 2 min), PCR amplification (35 cycles: 94°C/15 sec for denaturation, 58°C/30 sec for annealing and 68°C/3 min for extension) and final extension (1 cycle: 68°C/7 min). For H5N1/VSVRI, the cDNA generated using the Uni-12 primer [38] was used as a template. The mutibasic cleavage site (PQGEGRRKKR/GLF) in the H5/VSVRI was converted to a monobasic cleavage site (PQGETR/GLF) by amplifying the HA1 and HA2 separately using specific primers (Table 1). However, the NA segment of H5N1/VSVRI was amplified also using the universal primers [38] with *BsmB*I restriction sites (Table 1). Briefly, 2 μl cDNA were mixed with 5 μl 10× PCR Buffer, 1 μl 10mM dNTP mixture (0.2 mM each), 1.5 μl of 50 mM MgCl_2_ (1.5 mM), 2 μl of each Primer (40 pmoles) and 0.2 μl of Platinum Taq DNA Polymerase (10 U/μl) (Invitrogen, USA). The total volume was adjusted to 50 μl using nuclease-free water (Ambion, USA), followed by pre-denaturation at 94°C for 2 min, PCR amplification (35 cycles: 94°C/30 sec for denaturation, 58°C/30 sec for annealing and 68°C/3 min for extension), then final extension at 68°C/7 min. The PCR products were purified using “GeneJET Gel Extraction Kit” (Thermo Scientific, USA). The obtained RT-PCR fragments were next digested with *Bsa*I and B*smB*I for according to manufacturer protocol to be used further cloning.

**Table 1:** Primers used for the amplification of monobasic HA and the NA segments of H9N2/SA and H5N1/VSVRI.

**Segment**	**Primer**	**Sequence**
**H9**	Blunt-Ba-HA-F	TGATATCGGTCTCAGGGAGCAAAAGCAGGGGAATTTCTT
	Blunt-Ba-HA-R	TGATATCGGTCTCGTATTAGTAGAAACAAGGGTGTTTTTG
**H5**	Ba-LPAI-F1	TATAGGTCTCAGGGAGCAAAAGCAGGGGTT
	Ba-LPAI-Egy-R1	TATTGGTCTCT**GTCT**CTCCTTGAGGGCTATTTCTG
	Ba-LPAI-Egy-F2	TATCGGTCTCA**AGAC**GAGAGGACTATTTGGAGCTAT
	Ba-LPAI-R2	ATATGGTCTCGTATTAGTAGAAACAAGGGTGTTTTT
**N1 & N2**	Bm-NA-1	TATTCGTCTCAGGGAGCAAAAGCAGGAGT
	Bm-NA-1413R	ATATCGTCTCGTATTAGTAGAAACAAGGAGTTTTTT

• Underlined represents the sequences of the HA and NA segments which are compatible with the sticky ends of linearized vector.

•Underlined and **bold** represent the compatible internal sticky ends of HA1 and HA2 of H5N1/VSVRI to generate monobasic cleavage site.

### Generation of chimeric HA plasmids

In an attempt to circumvent the observed instability of the H9N2/SA-HA sequence, which we suspected to be located in the HA2-part, plasmids encoding chimeric HAs were generated by exchanging the HA1 sequence of the pHW2000 plasmid (pHW-HA-Bei) stably encoding the A/Chicken/Beijing/2/1997 (H9N2/Bei) HA with the corresponding sequence from the pSMART-LC-Kan plasmid (pSMART-HA-SA) possessing the HA-cDNA of A/chicken/Saudi Arabia/CP7/1998 (H9N2/SA) and *vice versa*. For this the HA1 sequences of pHW-HA-Bei and pSMART-HA-SA were excised using “FastDigest” *AlwN*I and *EcoR*I (Thermo Scientific, USA) for 15 min at 37°C and the HA1 sequence of the H9N2/Bei was cloned into pSMART-HA-SA, while the HA1 sequence of H9N2/SA was cloned into pHW-HA-Bei. To assure that plasmids to express the chimeric HAs have the same vector backbone the chimeric HA-cDNA in pSMART-HA-Bei/SA containing the HA1 of the H9N2/Bei-HA and HA2 of the H9N2/SA-HA was subcloned into pHW2000 after excision using *BsaI-*HF for 15 min at 37°C and ligation into *BsmB*I pre-cut pHW2000 using T4 ligase (Thermo Scientific, USA) at 22°C for 1 h. The resulting two pHW2000 plasmids (pHW-HA-Bei/SA, pHW-HA-SA/Bei) with the chimeric HAs (S1 Fig.) were transformed into competent XL-1 blue *E. coli* and analyzed for correct positive transformants.

### Construction of pMKP*ccd*B vector

To generate pMKP*ccd*B vector with *Bsa*I, *BsmB*I or *Aar*I cloning/restriction sites, Pol-I/*ccd*B/Pol-II cassettes containing the three different cloning/restriction sites were transferred from the respective pMP*ccd*B vector versions [39] using “*FastDigest*” *BsrB*I enzyme (Thermo Scientific, USA) at 37°C for 15 min. The gel-purified cassettes were ligated into the *EcoR*V-blunt ends of pSMART-LC-Kan vector using T4 DNA ligase (Thermo Scientific, USA) according to the manufacture instructions. The ligation reactions were then transformed into “One Shot ccdB Survival 2 T1R” competent *E. coli* cells (Invitrogen, USA) according to the manufacture instructions and cultured on LB agar plates. 16–18 h later, five individual colonies were picked up and cultured for 18 h at 37°C in 5 ml of LB media containing 30 μg/ml of Kanamycin (SERVA, Germany) followed by plasmid DNA extraction using mi-Plasmid Miniprep Kit (Metabion, Germany). The bacterial culture containing the correct plasmid DNA construct, confirmed by enzymatic digestion and sequencing, was subjected to large scale culture (250 ml LB media) and plasmid DNA extraction/purification using NucleoBond Xtra Maxi (Macherry-Nagel, Germany).

### Site directed mutagenesis

To silently mutate all *Bsa*I- and *BsmB*I-recognition sites in the backbone of pMKP*ccd*B, “Quickchange II XL Site-Directed Mutagenesis Kit” (Agilent Technologies, USA) was used as previously described [40]. The mutated forward and reverse primers (Table 2) were designed using PrimerX software package (*http://www.bioinformatics.org/*primerx/). Briefly, 1 μl of template plasmid DNA (2 ng) was mixed with 0.5 μl of each forward and reverse primer (2.5 pmoles/μl), 0.25 μl of 40 mM dNTP mix (10 mM each), 1.25μl “10× Pfu Ultra Buffer” and 0.25 μl of “Pfu Ultra Hotstart polymerase enzyme”. The total volume was finally adjusted to 12.5 μl with nuclease-free water (Ambion, USA). The reaction was kept at 95°C for 5 min followed by 18 cycles of 50 sec at 95°C for denaturation, 50 sec at 60°C for primer annealing and 5 min at 68°C for extension. The final extension was held at 68°C for 7 min. After the PCR program was finished, 0.25 μl of *Dpn*I (20 U/μl) was added directly to the reaction and incubated at 37°C for 60 min to digest the methylated and hemi-methylated parental strands. An aliquot of 2 μl of the final reaction was transformed into 20 μl “One Shot ccdB Survival 2 T1R” competent *E. coli* cells”. The correct clones were confirmed by restriction analysis and sequencing of the extracted plasmid DNA.

**Table 2:** Primers to mutate *BsmB*I and *Bsa*I sites in the genetic backbone of pMKP*ccd*B.

**Mutated Primer**	**Sequence**
***ccd*B-mut-F**	GCCAGTGTGCCGGTCT**T**CGTTATCGGGGAAGAAG
***ccd*B-mut-R**	CTTCTTCCCCGATAACG**A**AGACCGGCACACTGGC
**pMKP-KRGmut-F**	GGCGATCGCGTATTTCGTCT**A**GCTCAGGCGCAATCACG
**pMKP-KRGmut-R**	CGTGATTGCGCCTGAGC**T**AGACGAAATACGCGATCGCC
**pMPG2013-Ba-2 F**	CGACTCACTATAGGG**T**GACCCAAGCTGTTAAC
**pMPG2013-Ba-2 R**	GTTAACAGCTTGGGTC**A**CCCTATAGTGAGTCG

• Underlined and**bold** represent the point mutation to remove the *BsmB*I and *Bsa*I sites in the genetic backbone of pMKP*ccd*B.

### Cloning of HA- and NA-cDNAs from H9N2/SA and H5N1/VSVRI

An A-overhang was added to the purified HA- and NA- RT-PCR product of H9N2/SA and H5N1/VSVRI, which were then cloned into the T-overhangs of the classical cloning/sequencing vector pCR2.1 (Invitrogen, USA) as previously described [39]. The pCR2.1 constructs carrying the cloned NA-segments, proved by enzymatic digestion and subsequent sequencing, were digested by “FastDigest” *BsmB*I enzyme (Thermo Scientific, USA) at 37°C for 15 min, and then the NA-cDNAs were ligated into *BsmB*I-linearized pHW2000 using T4 ligase (Thermo Scientific, USA). The ligation reactions were transformed into DH5-alpha (New England Biolabs, USA) according to manufacture instructions.

Since the cloned HA-segments of H9N2/SA showed instability in the genetic backbone of pCR2.1 sequencing vector, the full-length RT-PCR products of HA segments amplified with primers containing *EcoRV* and *Bsa*I sites were digested with *EcoR*V enzyme (Thermo Scientific, USA) for 1 h at 37°C to produce blunt-ends to allow the ligation into the *EcoRV*-linearized pSMART-LC-Kan (Lucigen, USA). The positive and correct vector/insert constructs were digested with *Bsa*I-HF (NEB, Germany) for 15 min at 37°C. The digested product was then purified using “QIAquick PCR Purification Kit” (Qiagen, Germany). Meanwhile, the pHH21, pHW2000, pMP*ccd*B and pMKP*ccd*B were linearized with the “*FastDigest*” *BsmB*I (Thermo Scientific, USA) at 37°C for 15 min. The linearized vectors were then purified using “GeneJET Gel Extraction Kit” (Thermo Scientific, USA) and ligated to *Bsa*I-sticky ends of the amplified cDNAs using T4 DNA ligase (Thermo Scientific, USA) according to the manufacture instructions. The ligation products were transformed into Top10 (Invitrogen, USA), DH5-alpha (New England Biolabs, USA), DH10B (Invitrogen, USA), XL1-Blue (Stratagene, USA), Stbl2 (Invitrogen, USA), Sure (Stratagene, USA), Stbl3 (Invitrogen, USA), Stbl4 (Invitrogen, USA) and HB101 (Promega, USA) competent cells at different temperatures (25, 30 and 37°C) and various antibiotic concentrations (25, 50 and 100 μg/ml for ampicillin or 15 and 30 μg/ml for kanamycin). The extracted DNA of overnight individually propagated 5 colonies in LB media for each bacterial strain, temperature condition and RG system were analyzed using enzymatic digestion and sequencing.

### Chloramphenicol Acetyl Transferase (CAT) assay

To analyze quantitatively the polymerase II activity (mRNA synthesis) of pMKP*ccd*B in comparison with pHW2000, the viral ribonucleoprotein (vRNP) complex of influenza virus A/Hamburg/04/09(Hamburg/H1N1) was reconstituted *in vitro* using the according PB1 segment cloned either into pMKP*ccd*B or pHW2000. Briefly, 1 μg of pMKP*ccd*B or pHW2000 plasmids encoding the PB1 segment of Hamburg/H1N1 together with 2 μg pPOLI-CAT-RT [15] and 1 μg of pHW-PB2, 1 μg of pHW-PA and 2 μg of pHW-NP of Hamburg/H1N1 were transfected into 80-90% confluent monolayer of 293T cells using 2 μl “Trans-IT2020” (Mirus, USA)/1 μg DNA [15,41,42]. Cells were harvested 48 h post-transfection and then the cell extracts were prepared and tested for chloramphenicol acetyl transferase (CAT) activity (1:10 dilution) as described previously [15].

### Western blot analysis

Confluent 293T cells seeded in a 6-well plate, were transfected in triplicates with 2 μg plasmid DNA/well of pHW2000 and pMKP*ccd*B plasmids encoding the HA segment of H5N1/VSVRI (pHW-H5 HA/VSVRI and pMKP-H5 HA/VSVRI, respectively). Trans-IT2020” (Mirus, USA) was us as a transfection reagent as previously described above in “Chloramphenicol Acetyl Transferase (CAT) assay”. After 24 h, cells were lysed as previously described [43]. Cell lysates were separated on precast gradient NuPAGE Novex 4-12% Bis-Tris Protein Gels (Invitrogen, Germany) and transferred onto Immobilon-FL polyvinylidene fluoride (PVDF) membranes (Merck Millipore, Germany) AT 150 Volts/cm^2^. Following protein transfer, PVDF membranes were blocked using blocking buffer (1× TBS (20 mM Tris-HCl, pH 7.6, 140 mM NaCl) containing 5% non-fat dry milk) for 1 h at room temperature (RT). The membranes were washed 1 time using washing buffer (1× TBS-Tween: TBS containing 0.05% Tween20). Afterwards, detection of viral HA protein and cellular beta actin was achieved using primary mouse anti-influenza A virus (H5N1/HA1) monoclonal antibody (abcam, England) and rabbit anti-beta Actin monoclonal antibody (abcam, England) diluted in blocking buffer at a dilution of 1:5000 and 1:10000, respectively. One hour later, the membranes were washed for 3 times with washing buffer and incubated with secondary goat anti-mouse IRDye 800 and goat anti-rabbit IRDye 680 (1:10000 in blocking buffer containing SDS (1:1000 dilution of a 10% stock solution)), and added to the membranes in the dark for 1 h. After 3 times washing with washing buffer and one time with 1× TBS, the proteins were visualized using an “Odyssey Infrared Imaging System” and the according application software package (LI-COR, Germany). To quantify the amount of the protein, basic “Quantity One” software was used (Bio-Rad, Germany).

### Transfection and rescue of recombinant viruses

To generate the different prototype IVVS, 1 μg of each pHW2000- or pMKP*ccd*B plasmid encoding HA and NA segments of H9N2/SA or H5N1/VSVRI together with pHW2000 plasmids encoding the remaining segments of influenza virus A/Puerto Rico/8/34 (PR8, H1N1) were co-transfected into a co-culture of 293T/MDCK-II cells (ratio 3:1, 10 cm^2^) as previously described with minor modifications [14,39,44]. Briefly, the transfection mixture consisting of 180 μl “Opti-MEM” (Gibco, Invitrogen, USA) and 8 μg of plasmid DNA (1 μg of each plasmid) along with 16 μl “Trans-IT2020” (Mirus, USA) were incubated for 45 min at RT. The transfection mixture was then diluted to 1 ml using Opti-MEM and transferred to 80–90% confluent cell monolayer to allow transfection. The cells were then incubated for 8 h at 37°C in the presence of 5% CO2. The transfection medium was replaced with 1 ml of infection medium composed of “Opti-MEM” containing 100 I.U./ml penicillin, 100 μg/ml streptomycin and 0.2% BSA (PAA, Germany) and the cell cultures were incubated for another 12 h. Afterwards, an additional 1 ml of infection medium containing 2 μg/ml TPCK-treated trypsin was added (Sigma-Aldrich, USA). The cell culture supernatant was harvested 48 h after addition of the TPCK-treated trypsin and cell debris was removed by centrifugation at 2500 rpm for 5min at 4°C. An aliquot of 500 μl of each supernatant was used for the inoculation of fresh MDCK-II cells (25 cm^2^) and the cultures were then incubated for 72 h in the presence of TPCK-treated trypsin (1 μg/ml). To detect and characterize the recovered viruses, standard HA assay as well as RT-PCR using subtype-specific HA primers was performed (primers not shown). The titration of the recombinant viruses was performed using foci assay as described previously [45].

### Virus growth curves

To investigate the growth kinetic of the prototype IVVS expressing HA and NA of H5N1/VSVRI and H9N2/SA that were either generated using pMKP*ccd*B or pHW2000 reverse genetic systems, confluent monolayers of MDCK-II cells (3.5 cm dishes) were infected in triplicates with an multiplicity of infection (MOI) of 0.01 for 1 h at RT to allow virus adsorption. Unabsorbed virus was removed by washing the cells with 1× PBS^++^. Then, 2 ml infection medium (1× DMEM containing 100 I.U/ml penicillin, 100 μg/ml streptomycin, 0.2% BSA and 1 μg/ml TPCK-treated trypsin) was added. An aliquot of 200 μl from the supernatant was collected and replaced with 200 μl of fresh infection medium at 12, 24, 36 and 48 h post-infection (p.i.). Aliquots collected from the different viruses were then titrated using standard hemagglutinin assay and focus assay [45,46].

## Results

### Instability of cloned HA-cDNA of H9N2/SA and H5N1/VSVRI

**Figure 1:**
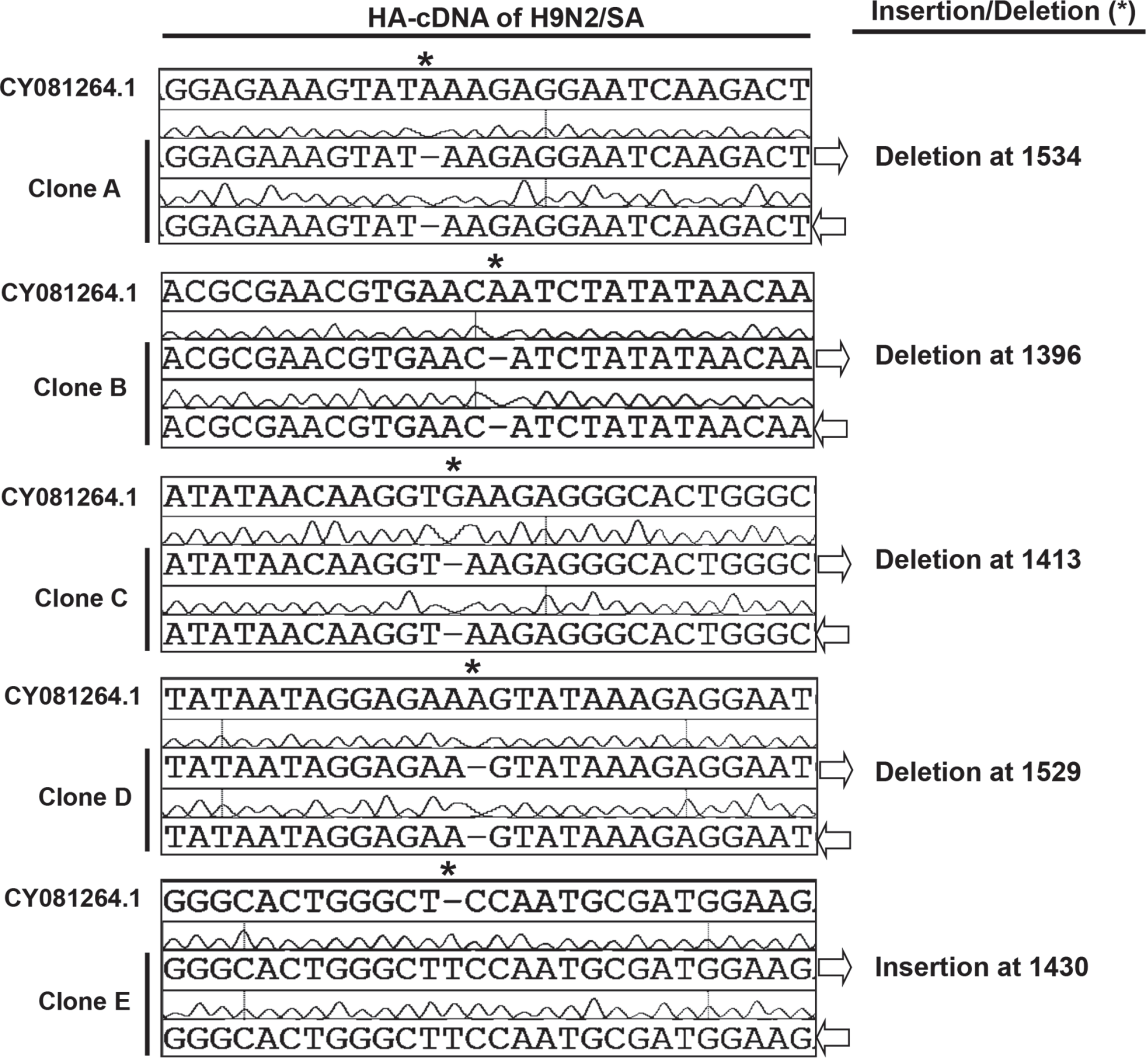
Instability of the H9N2/SA HA-DNA in standard pHW2000 and pHH21 reverse genetic vectors. Sequencing data of the H9N2/SA HA-RT-PCR product showed nucleotide deletions (**Clone A-D**) and insertions (**Clone E**) when aligned with the reference sequence of influenza A virus (A/chicken/Saudi Arabia/CP7/1998, H9N2, GenBank accession number: CY081264.1) indicated by boxed areas. Arrows (grey) indicate direction of overlapping sequences obtained with forward and reverse sequencing primers.

To generate the IVVS, the cDNA of HA and NA segments of the candidate strain have to be precisely and correctly cloned into a suitable reverse genetic (RG) system. Herein, the NA-cDNAs of H9N2/SA and H5N1/VSVRI were cloned into pCR2.1 for sequencing and further sub-cloning into the standard RG vector pHW2000. Both HA-cDNAs of H9N2/SA- and H5N1/VSRVI cloned in the backbone of pCR2.1showed genetic alterations. Upon transformation of DH5-alpha at 37°C none of the transformants contained the correct vector/insert construct as demonstrated by the enzymatic digestion (data not shown). Furthermore, repeated trials to transform pCR2.1 constructs with the respective HA-cDNAs into different *E. coli* strains (Top10, DH10B, XL1-Blue, Sure, Stbl2 and Stbl4) failed to obtain the correct plasmid constructs at both the standard temperature of 37°C and at lowered temperatures of 30 and 25°C and different antibiotic concentrations (25, 50 and 100μg/ml ampicillin). However, all transformants analyzed contained the correct vector/insert DNA when the same cDNAs were cloned into the cloning vector pSMART-LC-Kan. Therefore, the insert DNA of these positive clones carrying the HA-cDNA of H9N2/SA and H5N1/VSVRI in pSMART-LC-Kan, confirmed by enzymatic digestion and sequencing, were subsequently sub-cloned into the RG vectors pHW2000 [44], pMP*ccd*B [39] and pHH21 [14] and transformed into different competent bacterial strains (Top10, DH5-alpha, DH10B, XL1-Blue, Sure, Stbl2, Stbl3 and Stbl4), propagated at different temperatures (25, 30°C and 37°C) and antibiotic concentrations (25, 50 and 100 μg/ml ampicillin). To our surprise, both sub-cloned HA-cDNAs were unstable again under these conditions in the different vectors and bacterial strains. The enzymatic digestion of different constructs encoding both cDNAs did not match the expected pattern (data not shown). Although, a few clones of pHW2000, pMP*ccd*B and pHH21 carrying the HA-cDNA of H9N2/SA were demonstrated positive by enzymatic digestion, the sequencing results showed that they were not functional due to an insertion or a deletion of one nucleotide in the HA2 region that would lead to frame-shifting and hence inactive protein expression (Fig. 1).

### Chimeric H9-HA encoding-RG vectors

In comparison with the genetically stable HA segment of A/Chicken/Beijing/2/1997, H9N2 (H9N2/Bei), the HA1 region of H9N2/SA-HA shows a sequence homology of 88.5%, while the homology of the HA2 regions is 98.2%. Since the nucleotide deletion/insertions appeared in the HA2 (Fig. 1) after transformation of different RG vectors encoding the H9N2/SA HA-cDNA, we thought to generate a chimeric HA gene comprised of the H9N2/SA-HA1 region (containing the important antigenic sites for the generation of neutralizing antibodies [47]) ligated to the H9N2/Bei-HA2 region. By this approach we aimed to obtain a stable plasmid to create a recombinant vaccine virus expressing the desired epitopes [47]. To this point, two chimeric HAs were constructed—pHW-HA-SA/Bei and pHW-HA-Bei/SA (control), cloned into pHW2000 and transformed into XL-1 blue. The first chimeric HA segment was composed of the HA1 region of H9N2/SA and the HA2 region as well as the 3′ and 5′ non-coding regions (NCR) of H9N2/Bei (S1A Fig.). Under investigation the sequence of this chimeric HA also showed insertion/deletion instability in the HA2 region. In contrast, the sequence of the other chimeric HA possessing the HA1 of H9N2/Bei and the HA2 region as well as the 3′ and 5′-NCR of H9N2/SA (S1B Fig.) was genetically stable. Based on this finding, we favor that the instability of the cloned HA of H9N2/SA-cDNA is probably related to the sequence of the HA1 region, but its impact appears in the sequence of the HA2 region, preventing the use of the chimeric HA constructs.

### Efficient cloning and high genetic stability of HA segments upon transformation of vector-DNA into HB101 competent cells

**Figure 2:**
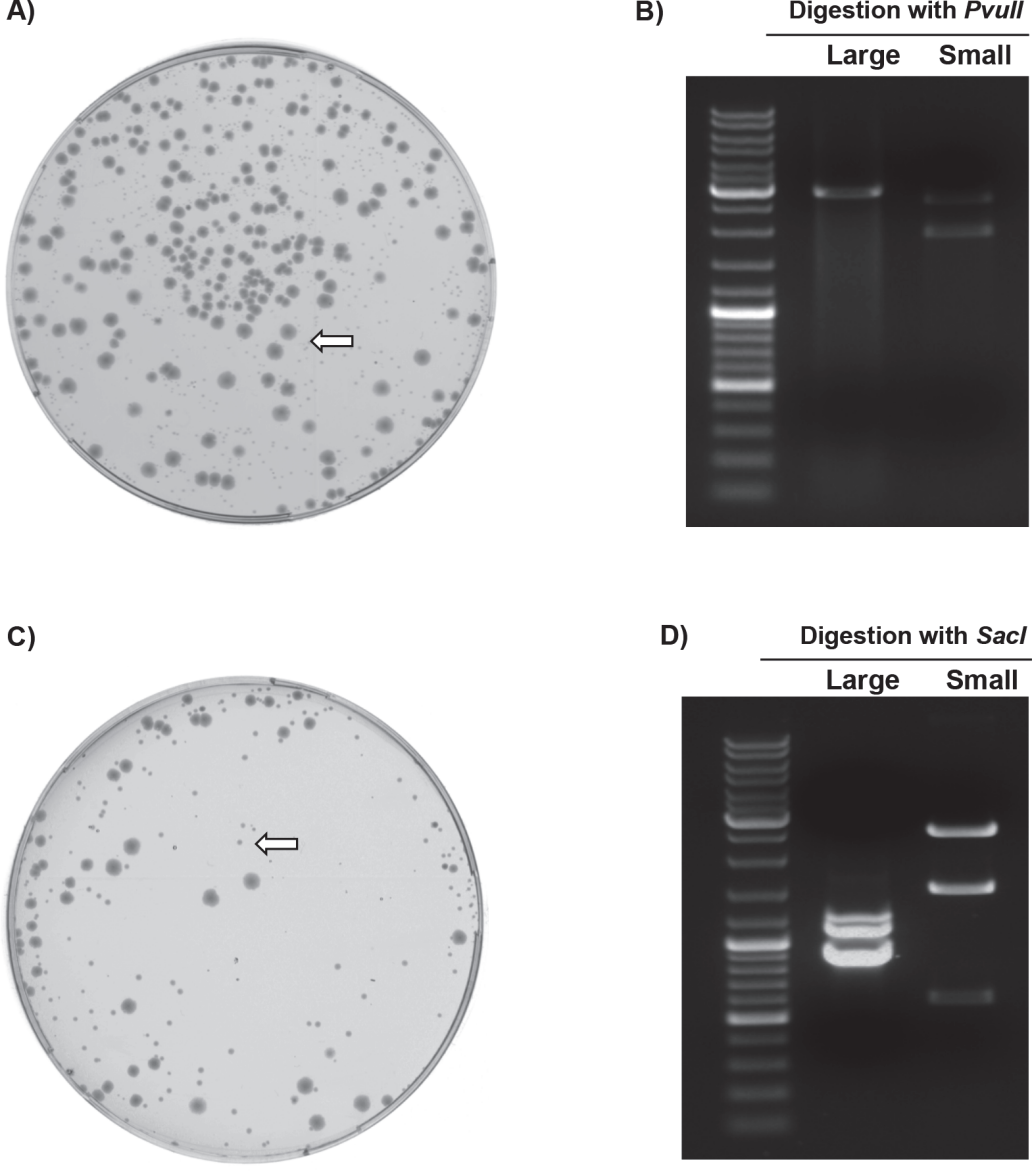
Transformation of the H9N2/SA-HA and H5N1/VSVRI-HA cloned in standard pHW2000 reverse genetic vector using *recA*13/HB101. A) and **C)**: Colonies with large and small phenotype were detected (arrow indicating the small type) after transforming the *recA*13/HB101 with pHW2000 encoding the DNA insert of H9N2/SA- or H5N1/VSVRI-HA segment, respectively. **B)** and **D)**: Restriction analysis of pHW-H9N2/SA-HA and pHW-H5N1/VSVRI-HA plasmid DNA isolated from the HB101 transformants showed the expected digestion pattern only with small-sized colonies and an incorrect pattern with all large-sized colonies.

Since different *E. coli* genotypes with *recA1* (DH5-alpha, DH10B, Top10 and Stbl4), *recA*-deficient (XL-1 blue), *recB*-deficient (Sure competent cells) or *recA13/*HB101-derived (Stbl3) failed to maintain the insert stability, we sought to transform the ligation reaction of pHW2000, pMP*ccd*B or pHH21 with the HA-cDNA of H9N2/SA into *recA*13/HB101 competent cells. Notably, the HB101 could faithfully maintain insert stability. Following transformation, two phenotypes of the transformants were detected on the agar plates, large and small colonies (Fig. 2: A and C). Only transformants of the small-sized colonies showed the expected restriction digestion pattern (Fig. 2: B and D) as well as the correct nucleotide sequence even under commonly used temperature condition and antibiotic concentration (37°C, 100 μg/ml ampicillin). Nevertheless, unlike the transformants of large-sized colonies containing incorrect plasmids, the transformants of small-sized colonies containing stable plasmids only grew poorly, probably to restrain the expression of an aberrant toxic protein product. However, not only toxic gene products, but also other mechanisms or bacterial pathways affecting recombination and/or removal of toxic sequences could account for the genetic instability. To confirm the advantageous ability of HB101 to stabilize the vector/insert DNA, also the previously unstable HA-cDNA of H5N1/VSVRI cloned into pHW2000 vector was transformed into HB101 competent cells. Similarly, the small-sized HB101 transformants harbored the correct vector/insert constructs. In a trial to ensure that this reduction in colony size is related to the cloned HA-cDNA of H9N2/SA and H5N1/VSVRI, the HB101 cells were also transformed with stable pHW-HA-Beijing and pHW-HA-KanI (pHW2000 plasmids encoding the HA segment of A/Chicken/Beijing/2/1997(H9N2) or A/Thailand/1(KAN-1)/2004(H5N1)). The pHW-HA-Beijing and pHW-HA-KanI transformants showed homogenous common large-sized colonies (S2 Fig.). This demonstrates that stability of genetic volatile inserts is probably maintained in HB101 by reduced bacterial growth.

### Low copy number pMKP*ccd*B vector stabilizes HA-cDNAs of H9N2/SA and H5N1/VSVRI

**Figure 3:**
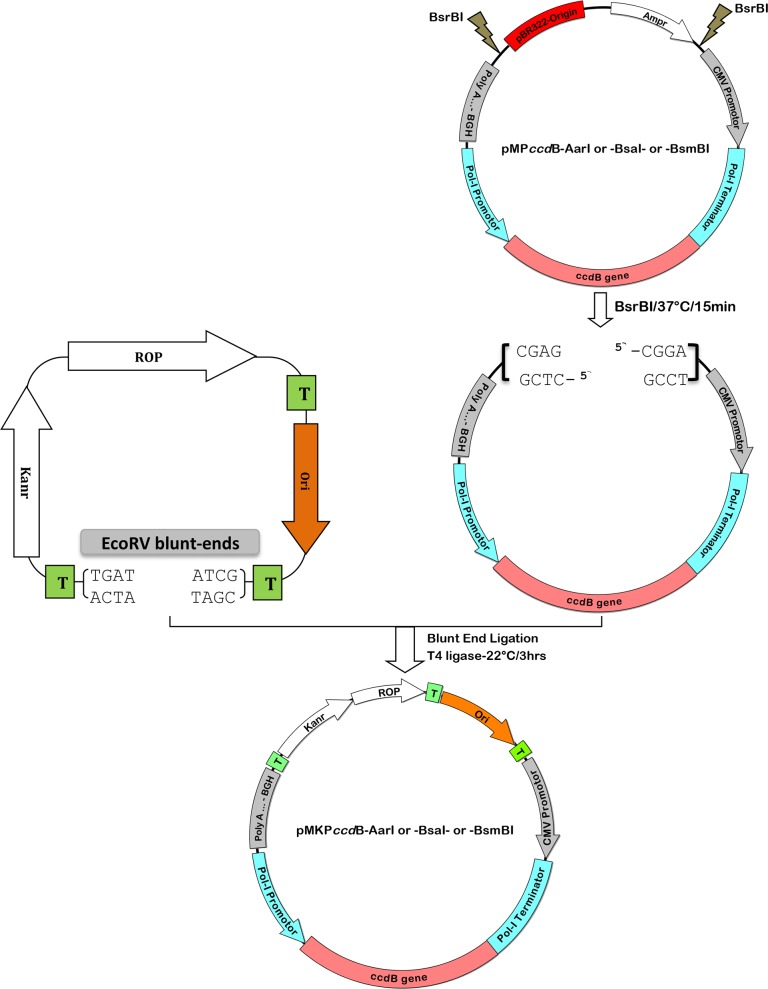
Construction strategy of the low-copy pMKP*ccd*B reverse genetic vector to clone unstable HA segments of IAV. The resulting vector is a hybrid of pSMART-LC-Kan and the Pol-I/*ccd*B/Pol-II cassette of pMP*ccd*B with either *AarI* or *Bsa*I or *BsmB*I sites for restriction/cloning of the desired insert. Briefly, the sequence representing the Pol-I/*ccd*B/Pol-II cassette excised from pMP*ccd*B by digestion with *BsrB*I were blunt end phosphorylated, and ligated into the *EcoR*V linearized pSMART-LC-Kan plasmid to generate pMKP*ccd*B. The pMKP*ccd*B was suitable to clone and maintain the genetically unstable RT-PCR product of H9N2/SA- and H5N1/VSVRI-HA. Transcriptional terminator (T); Repressor of Primer (ROP); low copy origin of replication (Ori); kanamycin resistance gene (Kanr), ampicillin resistance gene (Ampr).

Based on the fact that the H9-HA-cDNA was stable in the backbone of pSMART-LC-Kan and on the previous study of Zhou *et al.,* regarding the cloning of an unstable PB2 segment of A/Turkey/Ontario/7732/1966 (H5N9) into pHH21[36], we replaced the backbone of pMP*ccd*B [39] by pSMART-LC-Kan to construct an improved pMKP*ccd*B (Fig. 3). The improved vector also contains the *ccd*B gene as a negative selection marker, which helps to ameliorate the screening process [39,48]. Furthermore, all *BsmB*I- and *Bsa*I-sites in the backbone of pMKP*ccd*B vector were masked by silent mutations and the final construct was generated in three versions with *Aar*I, *BsmB*I or *Bsa*I cloning sites.

Although the H9N2/SA and H5N1/VSVRI HA-cDNAs cloned into the established pHH21, pHW2000, pMP*ccd*B vectors were not stable in a wide range of *E. coli* strains except for the recA13/HB101, the same cDNAs showed high stability when cloned into the improved pMKP*ccd*B vector and transformed into commonly used DH5-alpha, DH10B and XL-1 blue competent cells. This stability was illustrated by enzymatic digestion and verified by correct nucleotide sequencing (data not shown). To prove that the pMKP*ccd*B is fully functional in regard to the rescue of recombinant IAV by RG, recombinant virus was generated using pMKP*ccd*B plasmids encoding the entire eight viral segments of H9N2/Bei (data not shown).

**Figure 4:**
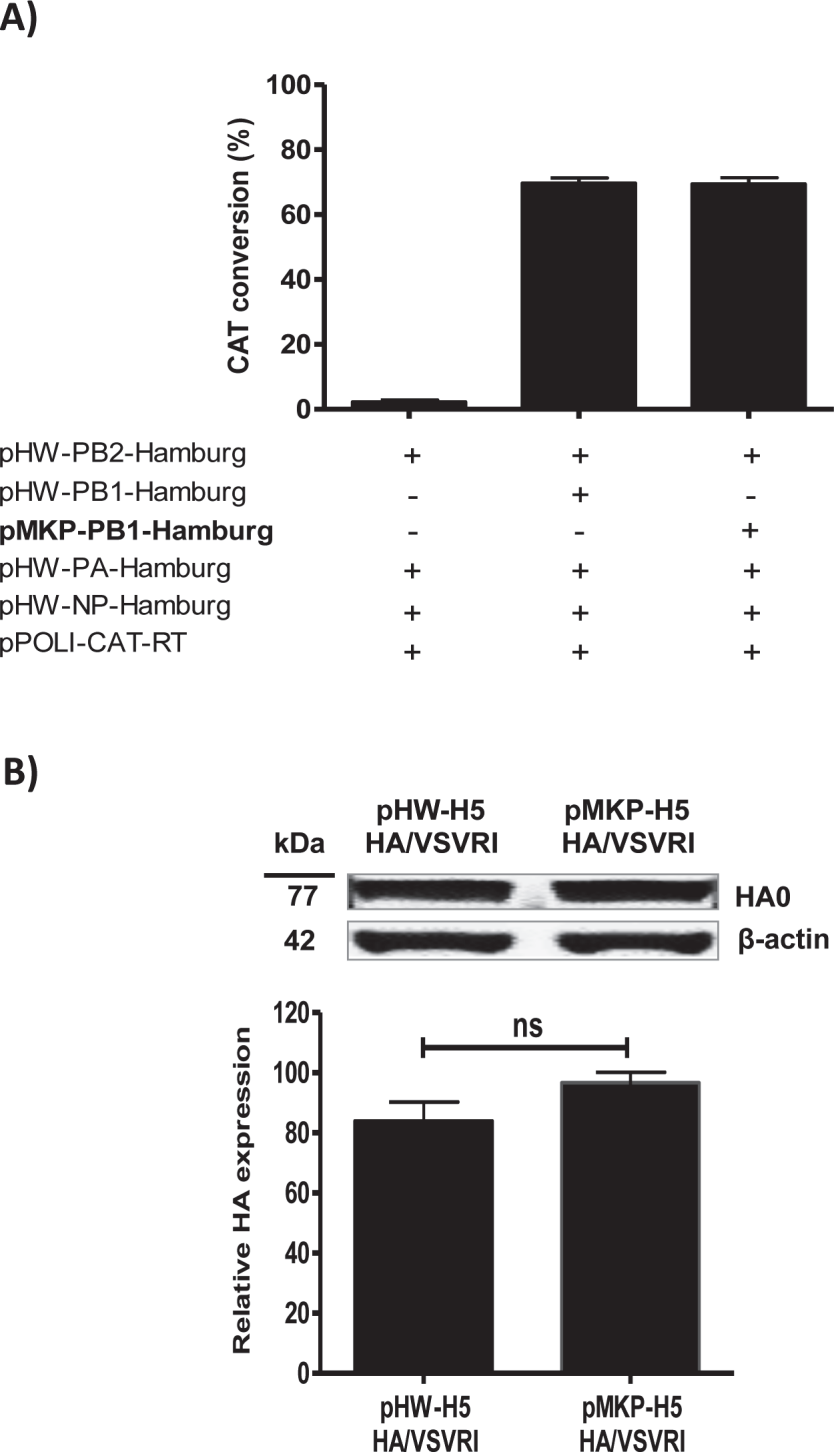
Effect of pSMART-LC-Kan cassette on the transcription efficiency of pMKP*ccd*B versus pHW2000 in eukaryotic cells. **A)** CAT activity in 293T cells: cells were transfected with pPOLI-CAT-RT plasmid and pHW2000 plasmids encoding PB2, PA and NP of A/Hamburg/04/09 (Hamburg/H1N1) together with pHW2000 or pMKP*ccd*B encoding PB1 segment of the Hamburg/H1N1. 24 h post transfection, cell extracts were prepared and assayed for CAT activity with [^14^C] chloramphenicol and thin-layer chromatography as previously described [15]. **B)** Western blotting analysis of expressed HA protein from H5N1/VSVRI: 293T cell monolayers were transfected in triplicates with pHW2000 or pMKP*ccd*B plasmid DNA encoding the HA segment of H5N1/VSVRI (pHW-H5 HA/VSVRI and pMKP-H5 HA/VSVRI, respectively). 24 h post transfection, cells were lysed, subjected to SDS-PAGE, transferred onto PVDF membrane and detected by HA-specific antibodies. The amount of HA protein expressed by pMKP-H5 HA/VSVRI plasmid was similar to that expressed by pHW-H5 HA/VSVRI. Cellular beta actin was used for normalization. Error bars show standard deviation (SD) of the mean (N = 3). (ns) indicates a non-significant difference (P value<0.05).

To further test whether the backbone of the vector, would affect the Pol-II transcription efficiency of pMKP*ccd*B we cloned the PB1 segment of influenza virus A/Hamburg/04/09 (Hamburg/H1N1) into pMKP*ccd*B (pMKP-PB1-Hamburg) or pHW2000 (pHW-PB1-Hamburg, control). These were transfected into 293T cells together with pHW2000 plasmids containing the cloned PB2-, PA- and NP-segment of Hamburg/H1N1 expressing the according mRNAs and pPol-I-CAT-RT to generate a vRNA-like Pol-I-transcript encoding the chloramphenicol acetyl transferase (CAT) reporter gene in order to reconstitute viral ribonucleoprotein complexes (vRNP) expressing CAT [15]. When analyzing the CAT activities of the vRNPs reconstituted with either pMKP-PB1-Hamburg or pHW-PB1-Hamburg we found that they were comparable (Fig. 4A).

Furthermore, the HA protein expression level of pHW2000 and pMKP*ccd*B encoding the HA of H5N1/VSVRI (pHW-H5 HA/VSVRI and pMKP-H5 HA/VSVRI, respectively) was determined in eukaryotic 293T cells as a direct indicator of the efficiency of the Pol-II directed mRNA transcription. Confirming our results of the CAT activity derived from the plasmid based *in vitro* RNP reconstitution, the HA protein production level of pMKP*ccd*B was comparable to that of pHW2000 plasmid (Fig. 4B). These findings clarify that the pSMART-LC-Kan backbone of pMKP*ccd*B does not impair the function of the Pol-I/ccdB/Pol-II cassette derived from the pMP*ccd*B vector in eukaryotic cells.

### Recombinant IVVS (6+2) viruses rescue and growth curve kinetic

After we had demonstrated that we could generate genetically stable RG plasmids for the H9N2/SA-HA and the H5N1/VSVRI-HA, either with the help of the *E. coli* strain HB101 or with the new pMKP*ccd*B vector in common *E. coli* strains, we next aimed to rescue 6+2 reassortant IVVS with the according HA and NA of either H9N2/SA or H5N1/VSVRI and RG plasmids carrying the remaining segments of PR8.

**Figure 5:**
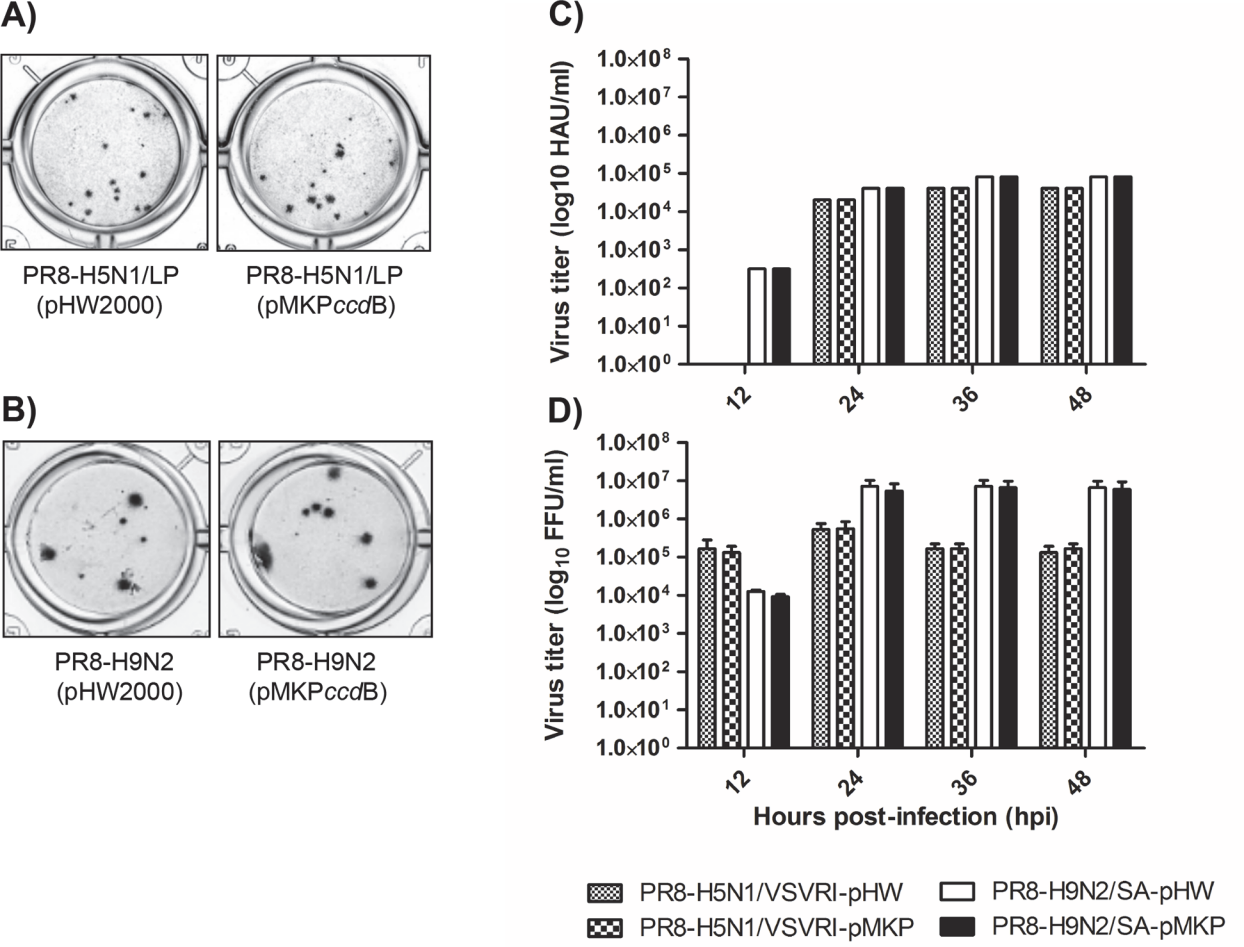
Characteristics of 6+2 reassortant viruses. The pMKP*ccd*B plasmid allowed the rescue of PR8-H9N2 and PR8-H5N1-LP generated by HA- and NA-cDNAs of H9N2/SA or H5N1/VSVRI (with Monobasic HA) cloned either into pHW2000 (transformed into HB101) or pMKP*ccd*B (transformed into DH5-alpha). The multibasic cleavage site of the H5N1/VSVRI-HA was mutated to a monobasic cleavage site. MDCK-II cells were infected with the reassortant viruses (MOI = 0.01) and allowed to replicate in media supplemented with1 μg/ml trypsin. Virus titers in the supernatants were shown 12, 24, 36 and 48 h p.i. in log10 HAU/ml **(C)** and log10 FFU/ml **(D)**. Shown is the foci morphology (**A, B**) and the growth kinetics (**C, D**) of PR8-H9N2 or PR8-H5N1 reassortant viruses. Error bars indicate SD of the mean (N = 3).

To attenuate the pathogenicity of the recombinant virus, the multi-basic cleavage site of H5-HA had been changed before to a mono-basic one [19,49–51]. The successfully rescued IVVS were detected with RT-PCR (S3A Fig.) and standard HA assay (S3B Fig.) and titrated using foci assay (Fig. 5: A and B). These IVVS generated by using the HA- and NA-cDNA cloned into the pMKP*ccd*B vector (PR8-pMKP-H9N2 and PR8-pMKP-H5N1/LP) were compared with the corresponding reassortant viruses rescued by using the pHW2000 vector and HB101 (PR8-pHW-H9N2 and PR8-pHW-H5N1/LP). Notably, similar replication characteristics were detected for PR8-pMKP-H9N2 compared to PR8-pHW-H9N2 and for PR8-pMKP-H5N1/LP compared to PR8-pHW-H5N1/LP as determined by standardized HA assay (Fig. 5C) and focus forming assay (Fig. 5D).

## Discussion

The RG approach has facilitated and accelerated the basic research on IAV as well as vaccine development in the past two decades [18,52,53]. To generate recombinant IVVS, the RT-PCR copies of the eight viral segments are cloned precisely into an RG system to produce vRNAs of the eight viral genomic segments and at least the mRNAs of the viral polymerase subunits (PB1, PB2, PA) and the nucleoprotein (NP). To obtain these vRNAs, the RT-PCR copies of the corresponding viral segments are cloned between an RNA polymerase I (Pol-I) promoter and terminator or the HDV ribozyme [13,14]. In order to express the necessary subunits of the viral polymerase and the NP that together with the vRNAs form the biological active RNPs, as well as the other viral proteins, the viral segments are also cloned between an RNA polymerase II (Pol-II) promoter and polyadenylation sequence [14,15,37]. The correct plasmids producing the vRNAs and mRNAs of IAV are then transfected together into suitable cells [54], to recover the recombinant IAV [14,44]. However, the occasional genetic instability of plasmids encoding the different viral genome segments (specially of HA and NA of candidate circulating or emerging IAV) in *E. coli* hinders a reliable and rapid construction of complete RG systems and the generation of IVVS. These are important pre-requisites to combat these strains and to assess their replication, virulence, pathogenicity, host range, and transmissibility. In this study, we demonstrate two different solutions to solve the stability problem of the cloned HA-segments from H5- and H9-subtypes transformed into different *E. coli* competent cells.

The plasmid vectors (for RG systems) are generally made from DNA sequences, derived from different sources such as bacteria and/or viruses and/or mammals. Occasionally, the foreign plasmid DNA can restrain or stop the growth of the bacterial host, undergoes structural rearrangement or can rapidly be lost during bacterial cell divisions [55]. Additionally, it is also well known that *E. coli* competent cells can introduce specific mutations and/or deletions in the foreign DNA to avoid the extensive aberrant expression of possibly toxic proteins and to maintain it in an inactive form. Therefore, different *rec*A mutations were generated to minimize the occurrence of such insertions, deletions and plasmid DNA rearrangement caused by the *rec*A protein of the bacteria [32].

Recently, several studies have demonstrated that vaccination with chimeric HA constructs can improve the antigen yield against IAVs and also elicit efficient polyclonal humoral responses to the HA ecto- and stalk domain protecting against heterologous and heterosubtypic challenges [56–58]. In our case, we thought that this approach might be helpful to overcome the problem of HA instability as we detected the bacterial artifacts in the stalk domain (HA2), but not in the globular head domain (HA1) of the H9N2/SA-HA. Nevertheless, based on our findings with chimeric HA constructs, that the origin of the observed instability was related to the sequence of the HA1 region, but its impact appeared in stalk domain (HA2), this strategy of chimeric HAs did not provide a solution to avoid the genetic instability of the cloned HAs.

To find an *E. coli* strain that would stably maintain the recombinant plasmids (e.g. pHH21, pMP*ccd*B and pHW2000) containing the HA-cDNAs of H9N2/SA and H5N1/VSVRI, we tested various *recA* alleles of standard competent bacteria; *recA1* (DH5-alpha, DH10B, Top10 and Stbl4), *recA*-deficient (XL-1 blue), *recB*-deficient (Sure competent cells) and *recA13* (HB101 and Stbl3). Unlike *recA1, recA*- and *recB*-deficient competent cells, the HA-RT-PCR products of H9N2/SA and H5N1/VSVRI were stably maintained in *recA*13/HB101 competent cells. The HB101 strain is a hybrid of *E. coli* K12 and B-bacteria, containing the *recA13* mutation that minimizes recombination and supports insert stability. In addition, it carries the hsdS20(r_B^−^_ m_B^−^_) restriction and methylation minus genotype, derived from *E. coli* B-bacterium, to prevent the cleavage of cloned DNA by endogenous restriction enzymes and allows efficient cloning of the methylated DNA [59]. In contrast, the various competent cells, which failed to stabilize the plasmids encoding HA-segments of H9N2/SA and H5N1/VSVRI are derivatives of the wild type *E. coli* K12 strain. Therefore, we think that the ability of HB101 to stably maintain the inserts is related to a combination of the *recA*13 mutation and the genomic sequence derived from *E. coli* B-strain. Despite the fact that the Stbl3 *E. coli* strain also accommodates the *recA*13 mutation and is derived from HB101, it did not stabilize the cloned HA-segments (data not shown). This strain was engineered to produce high yield of plasmid DNA and to enhance the cloning of methylated genomic DNA. It was also modified to optimally stabilize cloned products into lentiviral vectors [55]. Presumably, these modifications of the Stbl3 strain lead to a loss of the advantageous property of the parental HB101 strain. Importantly, highly unstable HA segments like the H9N2/SA-HA were stable in HB101 in small-scale and large-scale cultivations at 30°C. Nevertheless, in general the HB101 strain produces only low yields of plasmid DNA, which is also unprotected from restriction enzymes due to the restriction-modification minus phenotype (r^−^, m^−^).

Recently, the low-copy RG vector, pGJ3C3 was developed by substitution of the high copy number origin of replication (ori) from pUC in the standard unidirectional RG vector pHH21 with the low copy number ori of pSMART-LC-Kan (pBR322) [36]. Nevertheless, when we used the pHW2000, which also contains the low copy number ori (pBR322), the unstable HA constructs could not be stabilized in *recA*1 and *recA*-deficient bacteria. To this point, we constructed the bidirectional pMKP*ccd*B RG vector based our previously published pMP*ccd*B vector [39] and pSMART-LC-Kan to overcome the instability of the previously mentioned HA-cDNAs transformed into *recA*1 and *recA*-deficient competent bacteria. The improved RG system, pMKP*ccd*B, combines the main features of pSMART-LC-Kan and pMP*ccd*B. Several distinct elements derived from pSMART-LC-Kan ensure the maintenance of unstable inserts. Firstly, the repressor of primer (ROP) protein helps to down regulate and minimize the plasmid replication [60]. Secondly, the kanamycin selection is more stringent than ampicillin selection. Thirdly, the presence of three terminators derived from pSMART-LC-Kan prevents unwanted protein expression based on accidental promoter sequences present in the cloned genes. Despite the fact that cytomegalovirus (CMV) immediate-early promoter (derived from pMP*ccd*B) has the ability to initiate gene expression in *E. coli* [61], we assume that due to the low copy number pBR322 ori, the ROP gene and three termination sites of pMKP*ccd*B unwanted gene expression is prevented in *E. coli*. Furthermore, the change of the vector backbone of pSMART-LC-Kan did not affect the viral protein expression by the resulting pMKP*ccd*B in eukaryotic cells when compared with standard pHW2000 vector in RG systems.

Moreover, the pMKP*ccd*B has an efficient and strong negative selection marker, the *ccd*B gene, allowing only *E. coli* clones to grow that carry the plasmid with the inserted DNA [39,62]. To further improve the vector for an efficient one-step cloning procedure [63,64], the sites of the commonly used type-II restriction enzymes *Bsa*I and *BsmB*I, present in the pSMART-LC-Kan-derived backbone and in the *ccd*B gene (derived from of pMKP*ccd*B) were deleted. Ultimately, the low copy number pMKP*ccd*B RG vector containing the cloned cDNAs of the desired HA segments was successfully employed to reliably generate IVVS (6+2) for IAVs with indistinguishable replication characteristics compared to recombinant viruses engineered by the standard pHW2000 RG vector and HB101 strain. We should mention that our attempt to combine both strategies (transformation of HB101 with pMKP*ccd*B) only resulted in a poor plasmid DNA yield and therefore was not superior to transformation of standard E. coli (e.g. XL1-blue) with pMKP*ccd*B.

In summary, these improved strategies facilitate cloning and consequently the rescue of recombinant IAVs with unstable HA segments in the attempt to develop recombinant IVVS and will further allow basic research to focus on scientific questions regarding the biological characteristics of RG-derived recombinant IAVs.

## Supporting Information

S1 Fig HA chimeras. Schematic diagram of HA chimeras constructed to circumvent the observed instability of the H9N2/SA-HA sequence, which we suspected to be located in the HA2-part, by exchanging the HA1 sequence of the pHW2000 plasmid (pHW-HA-Bei) stably encoding the A/Chicken/Beijing/2/1997 (H9N2/Bei) HA with the corresponding sequence from the pSMART-LC-Kan plasmid (pSMART-HA-SA) possessing the HA-cDNA of A/chicken/Saudi Arabia/CP7/1998 (H9N2/SA) and *vice versa.* **A)** The chimeric HA segment of H9N2/Bei with the HA1 region of H9N2/SA cloned into pHW2000 (pHW-HA SA/Bei) showed genetic instability in the *E. coli* strain XL-1 blue. **B)** The chimeric HA of H9N2/SA with the HA1 region of H9N2/Bei cloned into pHW2000 (pHW-HA-Bei/SA) showed genetic stability in the *E. coli* strain XL-1 blue.
(TIF)

S2 Fig Colony morphology. Homogenous large-sized phenotype of colonies generated upon transformation of *recA*13/HB101 with **A)** pHW-HA-Beijing and **B**) with pHW-HA-KanI encoding the stable HA segment of A/Chicken/Beijing/2/1997(H9N2) or (A/Thailand/1(KAN-1)/2004(H5N1), respectively.
(TIF)

S3 Fig Identification of the rescued 6+2 reassortant viruses. The 6 + 2 recombinant viruses rescued by transfection of co-cultured 293T/MDCK cells with pMKP*ccd*B plasmids encoding PB2, PB1, PA, NP, M and NS genes of A/PR/8/34 (H1N1) and the HA (monobasic cleavage site) and NA genes of either A/chicken/Saudi Arabia/CP7/1998 (H9N2) or A/chicken/Egypt/VSVRI/2009 (H5N1), were analyzed by **A)** RT-PCR and PCR using specific primer pairs for HA and NA segments and by **B)** standard HA assay. As a template for RT-PCR and direct PCR, the purified viral RNA was extracted from the supernatants harvested 72 h post transfection. Unlike the PCR reactions, the RT-PCR reactions resulted in the expected HA and NA amplification products for both viruses, indicating that the products did not result from residual transfected plasmid DNA. This confirmed that the positive HA titer was related to the rescued viruses.
(TIF)
