# Supplementary figures and images for: Efficient Generation of Recombinant Influenza A Viruses Employing a New Approach to Overcome the Genetic Instability of HA Segments
Source: PLoS One. 2015 Jan 23;10(1):e0116917. doi: 10.1371/journal.pone.0116917 (PMC4304806; doi:10.1371/journal.pone.0116917)

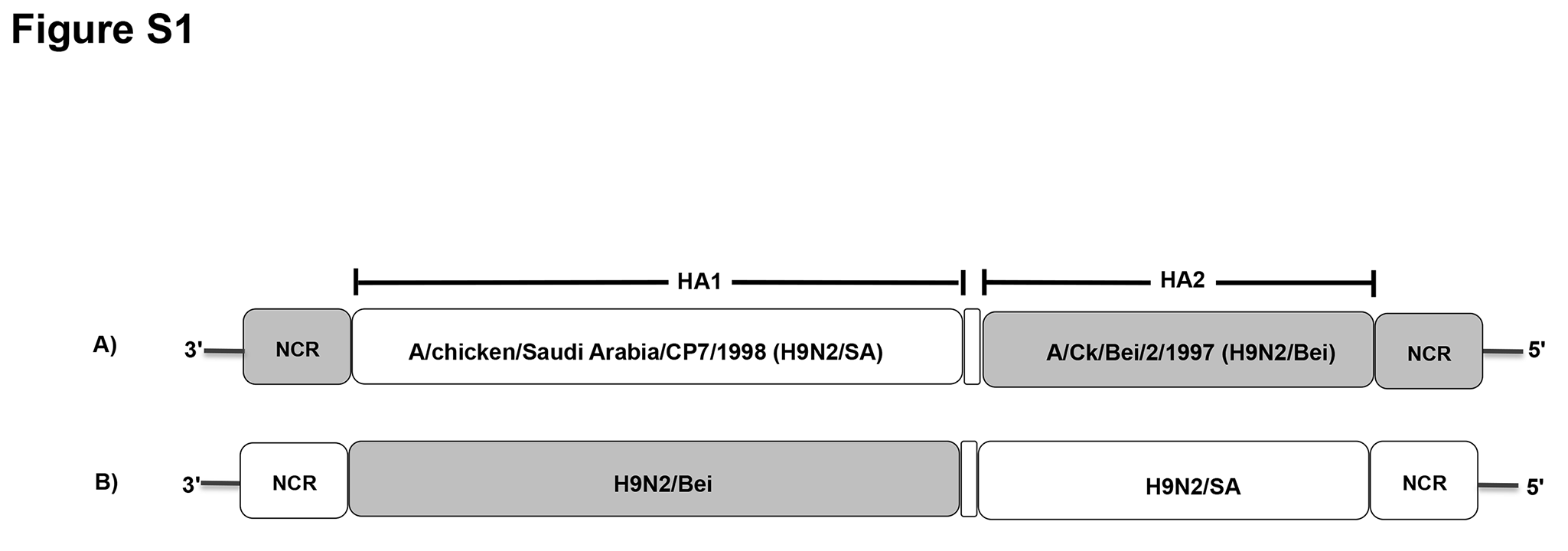

Supplement: S1 Fig — Schematic diagram of HA chimeras constructed to circumvent the observed instability of the H9N2/SA-HA sequence, which we suspected to be located in the HA2-part, by exchanging the HA1 sequence of the pHW2000 plasmid (pHW-HA-Bei) stably encoding the A/Chicken/Beijing/2/1997 (H9N2/Bei) HA with the corresponding sequence from the pSMART-LC-Kan plasmid (pSMART-HA-SA) possessing the HA-cDNA of A/chicken/Saudi Arabia/CP7/1998 (H9N2/SA) and vice versa. A) The chimeric HA segment of H9N2/Bei with the HA1 region of H9N2/SA cloned into pHW2000 (pHW-HA SA/Bei) showed genetic instability in the E. coli strain XL-1 blue. B) The chimeric HA of H9N2/SA with the HA1 region of H9N2/Bei cloned into pHW2000 (pHW-HA-Bei/SA) showed genetic stability in the E. coli strain XL-1 blue. (TIF) [file pone.0116917.s001.tif]

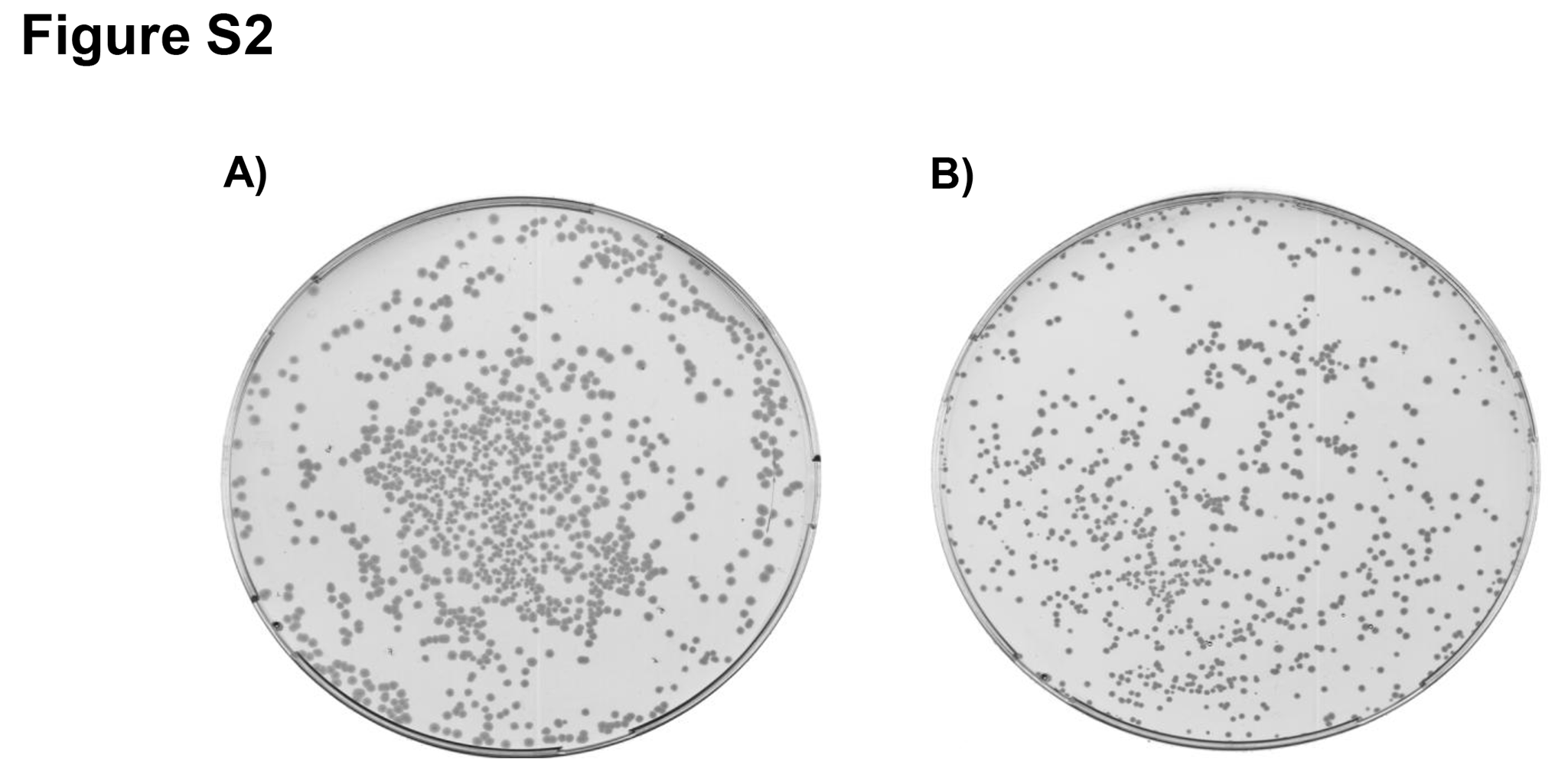

Supplement: S2 Fig — Homogenous large-sized phenotype of colonies generated upon transformation of recA13/HB101 with A) pHW-HA-Beijing and B) with pHW-HA-KanI encoding the stable HA segment of A/Chicken/Beijing/2/1997(H9N2) or (A/Thailand/1(KAN-1)/2004(H5N1), respectively. (TIF) [file pone.0116917.s002.tif]

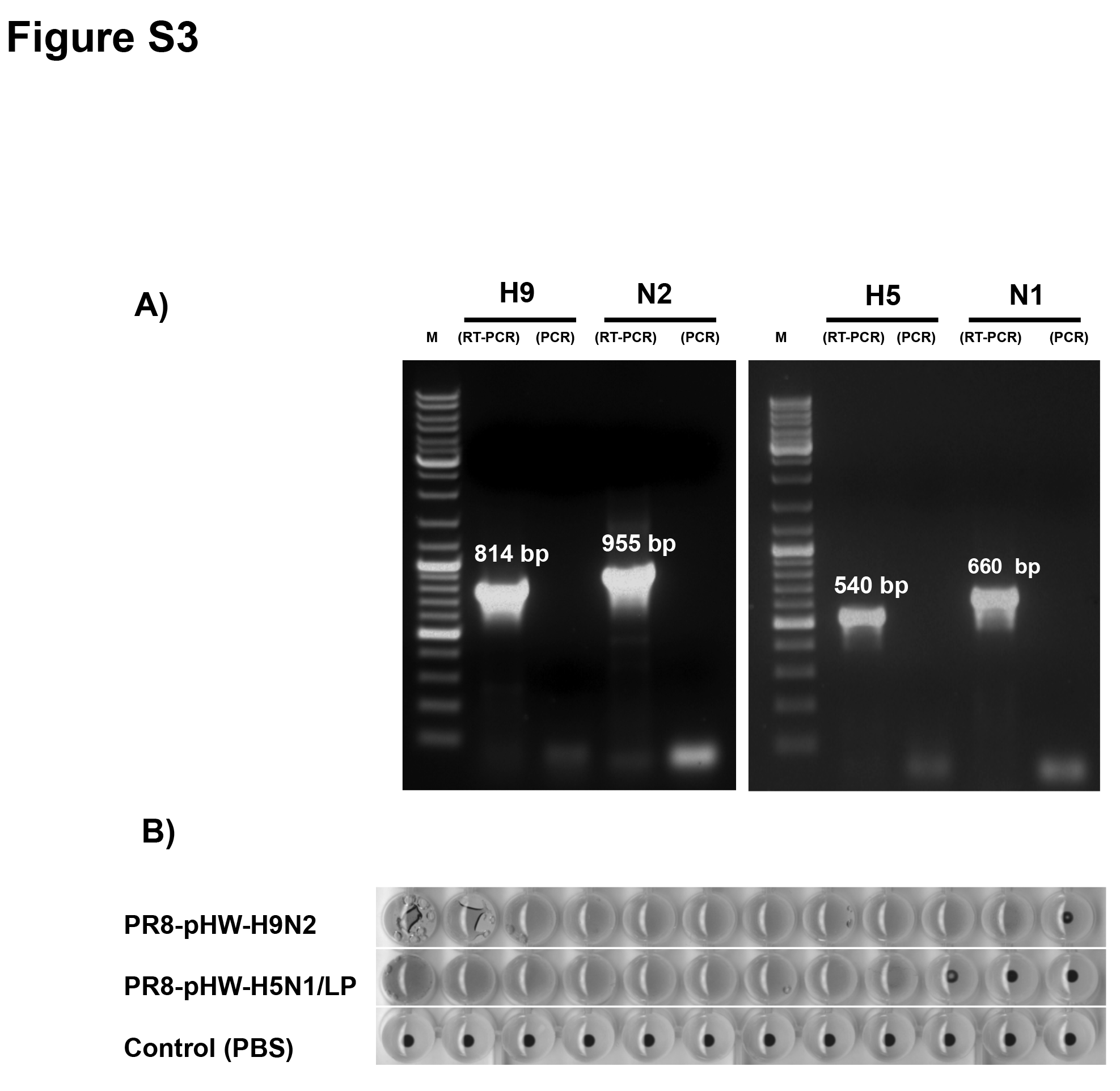

Supplement: S3 Fig — The 6 + 2 recombinant viruses rescued by transfection of co-cultured 293T/MDCK cells with pMKPccdB plasmids encoding PB2, PB1, PA, NP, M and NS genes of A/PR/8/34 (H1N1) and the HA (monobasic cleavage site) and NA genes of either A/chicken/Saudi Arabia/CP7/1998 (H9N2) or A/chicken/Egypt/VSVRI/2009 (H5N1), were analyzed by A) RT-PCR and PCR using specific primer pairs for HA and NA segments and by B) standard HA assay. As a template for RT-PCR and direct PCR, the purified viral RNA was extracted from the supernatants harvested 72 h post transfection. Unlike the PCR reactions, the RT-PCR reactions resulted in the expected HA and NA amplification products for both viruses, indicating that the products did not result from residual transfected plasmid DNA. This confirmed that the positive HA titer was related to the rescued viruses. (TIF) [file pone.0116917.s003.tif]
